# An Artifact of Perfluoroalkyl Acid (PFAA) Removal Attributed to Sorption Processes in a Laccase Mediator System

**DOI:** 10.1021/acs.estlett.3c00173

**Published:** 2023-03-24

**Authors:** Sophia D. Steffens, Edmund H. Antell, Emily K. Cook, Guodong Rao, R. David Britt, David L. Sedlak, Lisa Alvarez-Cohen

**Affiliations:** † Department of Chemistry, University of California, Berkeley, California 94720, United States; ‡ Department of Civil and Environmental Engineering, University of California, Berkeley, California 94720, United States; § Department of Chemistry, University of California, Davis, California 95616, United States

**Keywords:** PFAS, EPR, enzyme, sorption, mediator

## Abstract

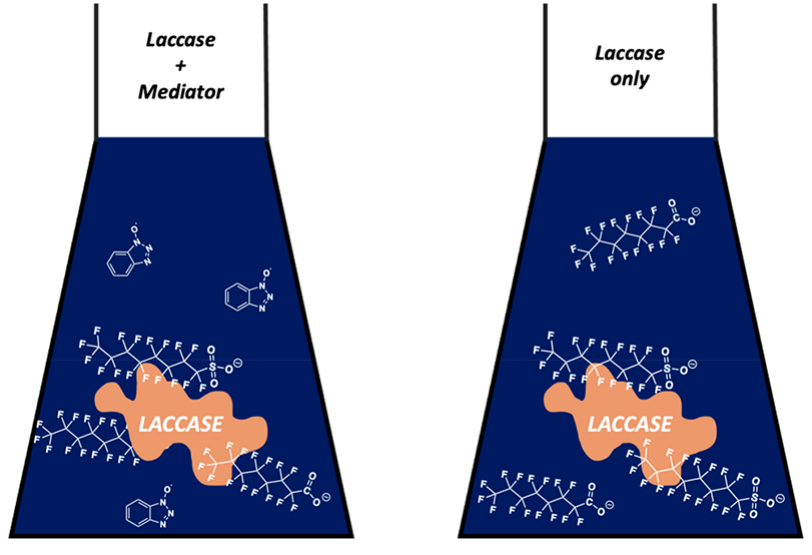

Fungi and laccase mediator systems (LMSs) have a proven track record of oxidizing recalcitrant organic compounds. There has been considerable interest in applying LMSs to the treatment of perfluoroalkyl acids (PFAAs), a class of ubiquitous and persistent environmental contaminants. Some laboratory experiments have indicated modest losses of PFAAs over extended periods, but there have been no clear demonstrations of a transformation mechanism or the kinetics that would be needed for remediation applications. We set out to determine if this was a question of identifying and optimizing a rate-limiting step but discovered that observed losses of PFAAs were experimental artifacts. While unable to replicate the oxidation of PFAAs, we show that interactions of the PFAA compounds with laccase and laccase mediator mixtures could cause an artifact that mimics transformation (≲60%) of PFAAs. Furthermore, we employed a surrogate compound, carbamazepine (CBZ), and electron paramagnetic resonance spectroscopy to probe the formation of the radical species that had been proposed to be responsible for contaminant oxidation. We confirmed that under conditions where sufficient radical concentrations were produced to oxidize CBZ, no PFAA removal took place.

## Introduction

Typical removal and destruction technologies for perfluoroalkyl acids (PFAAs) such as advanced oxidation and reduction processes, adsorption technology, and incineration are chemically and energetically intensive.^1^ Given the vast scale of contamination,^2^ the lower energy and chemical requirements of biological treatment strategies are an enticing, yet elusive, alternative.

Laccase enzymes, multi-copper oxidases found in plants, insects, fungi, and bacteria,^3,4^ have inspired biobased strategies for the treatment of recalcitrant compounds due to their ability to oxidatively degrade lignin using molecular oxygen as a terminal electron acceptor.^5^ Bacterial laccases tend to have a higher thermotolerance and a wider pH range,^6,7^ while fungal laccases tend to have higher redox potentials (∼0.5–0.8 V vs NHE).^4,8,9^ While they exhibit broad substrate specificity,^4^ the performance of laccase with respect to contaminant transformation is limited by its oxidation potential. Nonetheless, fungal laccases have been investigated in combination with low-molecular weight mediator compounds for their ability to oxidize target substrates that cannot be oxidized by laccase alone.^3,10−13^ This multistep oxidation cycle, in which laccase oxidizes a chemical mediator, the chemical mediator oxidizes a target substrate, and molecular oxygen is reduced to water, is termed the laccase mediator system (LMS).^10,12,13^

Fungal LMSs have been successfully applied to lignin degradation and biobleaching of kraft pulp^11−13^ and have gained interest for the treatment of recalcitrant water contaminants, including pesticides, pharmaceuticals,^14,15^ and, more recently, PFAAs.^16,17^ Researchers have reported the use of multiple white rot fungal laccase enzymes, including *Trametes versicolor*(14,15,18−20) and *Pleurotus ostreatus*,^9,16,17,21^ in combination with nitroxyl radical mediators for contaminant degradation. Although the electron transfer process in the delignification mechanism is well documented,^9,11,21−25^ the mechanism of degradation for non-lignin substrates has not been thoroughly investigated.

We sought to improve the scope and efficacy of LMS treatment of PFAAs by a mechanistic investigation of the “key players” in the multistep oxidation reaction. We tested multiple commercially available laccase enzymes, assessed enzyme activity, and evaluated the isolated enzyme mediator reaction by testing reactivity toward a proxy compound and confirming the generation of the nitroxyl radical mediator via paramagnetic resonance (EPR) spectroscopy. We found that the LMS could not achieve detectable degradation of PFAAs. However, in the process of teasing apart each key player’s role in the reaction, we observed an artifact in transformation studies of perfluorooctanoic acid (PFOA) and perfluorooctanesulfonic acid (PFOS) that suggests that laccase enzymes may remove PFOA and PFOS by sorption.

## Materials and Methods

### Chemicals

Commercial enzymes were purchased from MilliporeSigma (*Trametes versicolor* and *Agaricus bisporus*) and Creative Enzymes (native laccase, white rot fungi). 1-Hydroxybenzotriazole (HBT) was purchased from AnaSpec. *N*-Hydroxyphthalimide, violuric acid, TEMPO, and AZADO were purchased from MilliporeSigma. High-performance liquid chromatography-grade acetonitrile (CH_3_CN) was purchased from Fisher Scientific. Chemicals and enzymes were used without further purification. Mass-labeled internal standard compounds for PFAS analysis were purchased from Wellington Laboratories.

### Sampling and Extraction of Batch Reactors

Reactor solutions consisted of a target substrate in an appropriate buffer or copper solution [e.g., 50 mM sodium malonate (pH 4.5–5), 100 mM sodium acetate (pH 5), and 10 mM CuSO_4_ (pH 5)] to which a selected mediator and enzyme were added. Reactors were prepared in 20 mL glass scintillation vials with a 5 mL solution volume to ensure oxygenated headspace, unless described otherwise. Solutions were prepared with a buffer, a small volume of a concentrated substrate stock [e.g., 100 μL of aqueous PFOS, PFOA, or carbamazepine (CBZ)], a small volume of a concentrated mediator stock (e.g., 50–100 μL of HBT in CH_3_CN), and a laccase enzyme. Mediator-free control reactors were prepared with equal volumes of CH_3_CN. Reactors were placed in a floor shaker incubator (New Brunswick Excella E25) set to 30 °C and 130 rpm.

**Figure 1:**
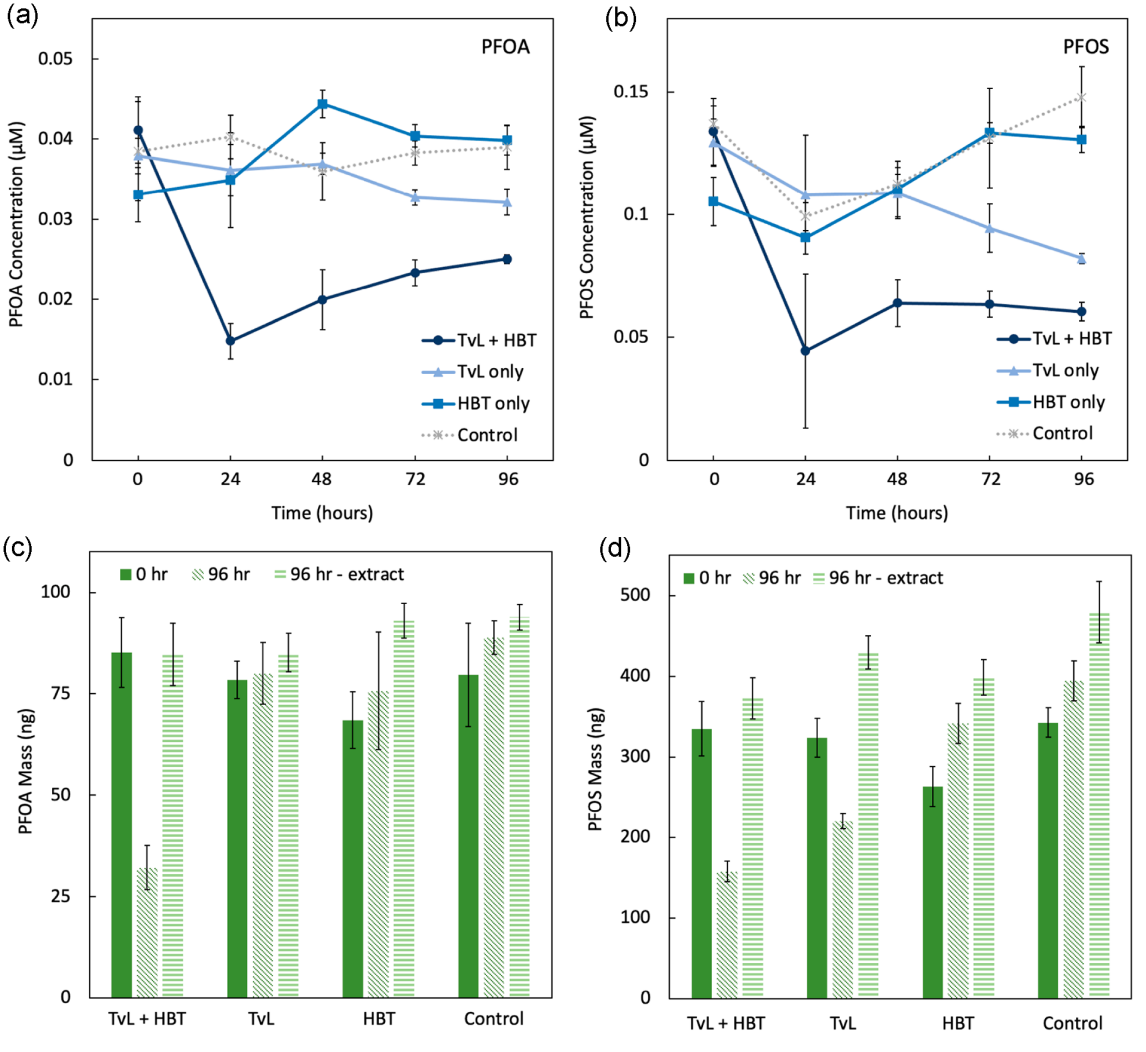
Reactors were amended with 1 unit/mL TvL and 1 mM HBT twice daily. Concentrations of (a) PFOA and (b) PFOS detected in an aqueous aliquot from the reactors. Masses of (c) PFOA and (d) PFOS in reactors calculated from the detected concentration and total solution volume at the beginning of the experiment, at the 96 hour time point, and in the extracted solution volume at the 96 hour time point. Error bars are the standard deviation of triplicate reactors.

**Figure 2:**
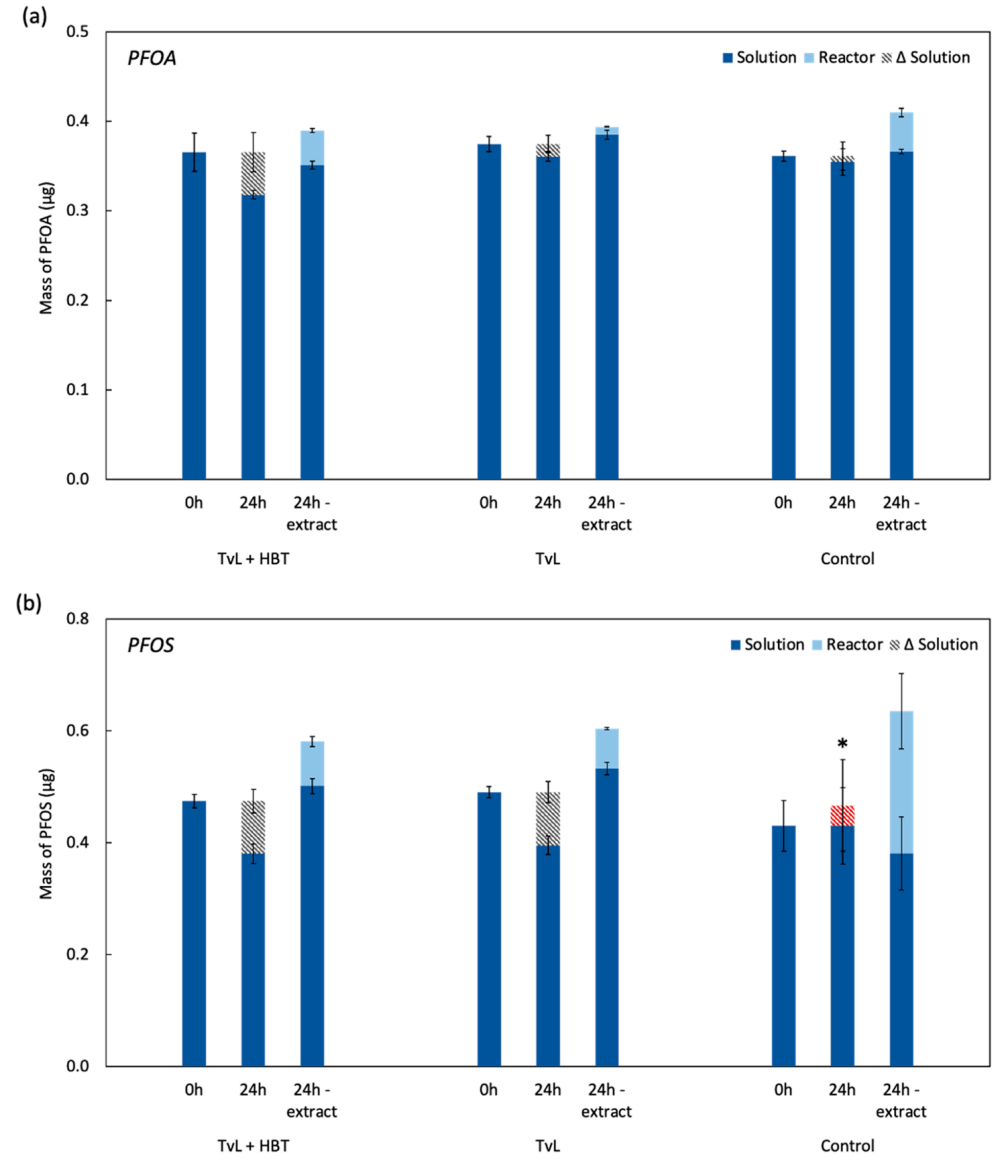
Reactors were treated with a single dose of 2 units/mL (60 mg) TvL and 2 mM HBT. Detected masses of (a) PFOA and (b) PFOS measured from a solution aliquot immediately after reactor preparation (0 h) and after incubation for 24 hours (24 h); Δsolution is calculated from the mass detected in the aliquot between 0 hours and 24 hours (i.e., the mass attributed to protein sorption). At 24 hours, the protein/buffer solution and the reactor were extracted separately with basic MeOH. The mass of PFAAs from the extracted solutions was measured for comparison. Error bars are the standard deviation of triplicate reactors. *Note that Δsolution for the PFOS control is negative and is plotted overlaying the 24 hour value.

At the appropriate daily or weekly sampling time, reactors were opened for a minimum of 10 minutes to promote aeration. Sample aliquots were removed from reactors and immediately diluted in basic methanol (0.5% ammonium hydroxide) to terminate the reaction. Reactor solutions and reactor vials were extracted with basic methanol at the end of the experiment. Full details of the extraction protocol can be found in Text S1 of the Supporting Information. In the extraction protocol used for the experiment summarized in Figure 1, basic methanol was added directly to the original reactor (2× dilution) after weighing the remaining solution volume. In a revised extraction protocol used for the experiment summarized in Figure 2, the enzyme/buffer solution was transferred to a fresh vial and diluted 2-fold with basic methanol; to the original reactor were added 10 mM CuSO_4_ and basic MeOH (4 mL total, 50/50 by volume) to recover the PFAA mass sorbed to the reactor walls. Samples were analyzed by liquid chromatography-tandem mass spectrometry (LC-MS/MS); details of the analytical method can be found in Text S2 of the Supporting Information. Statistical analysis was performed in Python 3.8.5 using the *SciPy* library.

### EPR Spectroscopy

EPR spectroscopy studies were performed at the CalEPR Center at the University of California, Davis. X-Band (9.4 GHz) continuous-wave (CW) EPR spectra were recorded on a Bruker Biospin EleXsys E500 spectrometer with a super high Q resonator (ER4122SHQE), an ESR900 liquid helium cryostat with a temperature controller (Oxford Instruments ITC503), and a gas flow meter. CW EPR spectra were recorded under slow-passage, nonsaturating conditions. Spectral simulations were performed in Matlab 2022b (Mathworks) with Easyspin 5.2.35 toolkit.^26^ Details of sample preparation for the EPR experiments can be found in Text S3 of the Supporting Information.

## Results and Discussion

Multiple laccase enzymes were screened with five nitroxyl radical mediators (Figure S1) for their ability to degrade PFOS. Because the white rot laccase *P. ostreatus* previously reported for PFAA degradation^16,17^ is no longer commercially available, we evaluated an alternative native white rot laccase (Creative Enzymes) and *A. bisporus* (Sigma), both of which are “high-redox” laccases capable of oxidizing nitroxyl mediator compounds.^27,28^ Upon subjecting solutions containing 1 μM PFOS and 10 mM CuSO_4_ to weekly amendments of 1 unit/mL enzyme and 20 μM mediator, we expected to see evidence of transformation within 20–30 days on the basis of the results in the *P. ostreatus*/HBT system that reported >20% and >30% removal at these respective time points.^17^ However, we observed no significant differences among the enzyme/mediator treatment conditions and the enzyme-only control (Figure S2).

We tested literature-reported conditions using *T. versicolor* laccase (TvL) and HBT to degrade CBZ,^15^ an antiepileptic drug and persistent, hydrophobic contaminant.^15,29^ We confirmed the reactivity of the TvL/HBT system and found that three doses of 2 units/mL TvL and 1 mM HBT over the course of 120 h resulted in significant removal, ∼35%, of CBZ (*p* < 0.001; two-tailed *t* test) (Figure S3). However, monitoring PFOA treatment with six doses of 1 unit/mL TvL and 1 mM HBT over the course of 2 weeks did not indicate significant removal compared to an untreated control (*p* = 0.29; two-tailed *t* test) (Figure S4), which was unexpected given that >20% PFOA removal after 10 days was previously reported.^16^

Considering that the retention of enzyme activity might control the reaction, we monitored enzyme activity in a sodium acetate buffer compared to that in a CuSO_4_ solution (Text S5 and Figure S5); the enzyme retained a similar activity profile in both. Furthermore, we confirmed the generation of the oxidation of HBT to the benzotriazole-*N*-oxyl (BTNO) reactive radical species (Figure S6) with EPR. Following a previous report,^30^ we generated the BTNO radical in a CH_3_CN solution by oxidizing HBT with Ce(IV) and in an aqueous solution by incubating TvL and HBT in the presence of CuSO_4_ (Figure S7). A hyperfine-split feature characteristic of the radical species^22^ is shown in the room-temperature spectrum in CH_3_CN but was not resolved in the EPR spectrum of the frozen aqueous solution, likely due to the line broadening caused by anisotropies in the latter.

Upon confirming BTNO radical generation, retention of enzyme activity, and the reactivity of the TvL/HBT system toward the proxy CBZ, we considered that slow reaction kinetics might have prevented observable transformation at the substrate concentrations initially tested (i.e., 1 μM PFOA and 1 μM PFOS). Therefore, we treated a set of parallel reactors containing either 0.1 μM PFOA or 0.1 μM PFOS in a 10 mM CuSO_4_ solution. Reactors were dosed twice daily because decreased enzyme activity was observed over the course of 6–8 h in the presence of HBT (Figure S8). Aliquots were removed daily and diluted directly in basic MeOH to determine PFOA and PFOS concentrations. Indeed, we observed decreases in measured PFAA concentrations at the 24 hour time point after two doses of the enzyme and mediator, specifically, a 64% decrease in PFOA concentration (Figure 1a) and a 67% decrease in PFOS concentration (Figure 1b).

At the end of the experiment (96 h time point), the reactors were extracted with basic methanol. Basic methanol was added directly to the reactor to dilute the aqueous solution by a factor of 2. Upon calculation of the total mass of PFOA or PFOS contained in the TvL/HBT-treated reactors at the first time point and the 96 h time point, 99 ± 14% of the PFOA mass and 111 ± 11% of the PFOS mass were recovered in the extracted solutions, suggesting that the decrease in concentration was likely due to physical phenomena (e.g., sorption) (Figure 1c,d). We considered that the “over-recovery” of both PFOA and PFOS, particularly in the control reactors, might be caused by fast sorption to the reactor that was not accounted for in the initial concentration measured from aliquots sampled at the beginning of the experiment. Prior studies have indicated low-energy barriers for the sorption of PFOA and PFOS to surfaces.^31^

To further evaluate the sorption phenomena and to differentiate the PFAA mass sorbed to the enzyme and the mass of PFAA sorbed to the reactor, we conducted an experiment in which we separately extracted the enzyme/buffer solution and the reactor (Text S1). Reactors containing either PFOA or PFOS (*∼* 0.1 μM) were treated with a single dose of enzyme and mediator (2 units/mL TvL and 2 mM HBT) or enzyme only (2 units/mL TvL). We increased the dose to match the total enzyme and mediator added within the first 24 h of the experiment summarized in Figure 1. Solutions were then incubated for 24 hours. Solution aliquots were removed upon reactor preparation (0 hours) and at the 24 hour time point and diluted directly in basic methanol. At the 24 hour time point, enzyme/buffer solutions and vials were extracted separately with basic methanol to determine mass loss due to enzyme–substrate sorption and reactor–substrate sorption (Figure 2).

We observed an apparent PFOA loss in the TvL/HBT-treated reactors of 18 ± 2% compared to the total PFOA mass extracted (Figure 2 and Table S2); for PFOS, we observed an apparent loss of 34 ± 4% compared to the total PFOS mass extracted (Figure 2 and Table S3). In the reactors containing only the TvL enzyme, we observed similar losses for PFOS of 35 ± 3% compared to the total PFOS mass extracted; for PFOA, however, a substantial mass loss was not observed. These results were consistent with the results presented in Figure 1. Upon extracting PFOS from the reactors, we observed that sorption of PFOS to the reactor was greater in the enzyme-free control (40 ± 12% of total mass) relative to the TvL/HBT (14 ± 2% of total mass) and TvL (12 ± 1% of total mass) treatments (Table S3), suggesting that sorption to the protein was more favorable. Sorption of PFOA to the reactor was more similar among the treatments and control (Table S2).

The difference in mass detected in the 0 and 24 hour subsample (Δsolution) can be reasonably attributed to enzyme sorption. For PFOA, a 13 ± 6% mass loss was observed in a solution treated with TvL/HBT, while a substantial mass loss was not observed in the TvL-treated or control reactors. For PFOS, a 20 ± 5% mass loss was observed in a solution treated with TvL/HBT, and a 19 ± 4% mass loss was observed in a solution treated with only TvL. As shown in Figure 2, the Δsolution mass (hashed bar) indicates a complete mass balance, within error, compared to the total mass extracted from the protein/buffer solution.

Both PFOA and PFOS have been shown to have a strong affinity for proteins, namely, human serum albumin (HSA)^32−34^ and bovine serum albumin (BSA).^35−37^ Proteins have in fact been investigated as sorbents for PFOA, including BSA, casein, egg white albumin, and lysozyme; it was found that these proteins could achieve ≤93% removal depending on the aqueous conditions. It was found that removal percentages for PFOA varied at different pHs, indicating that the charge of specific amino acid residues influenced the sorption affinity of PFOA.^37^

The affinity of PFAAs for BSA has also been shown to be influenced by PFAA chain length, and multiple binding mechanisms have been teased out, including binding at specific protein residues and nonspecific interactions (e.g., hydrophobic interactions).^36^ The study of BSA binding also indicated that PFOS could form multiple strong and weak associations with a single molecule of BSA, while shorter chain PFSAs and PFCAs showed lower affinity for BSA.

Our results indicated differences in the TvL and PFAA sorption mechanism with and without HBT, which alters the protein structure upon oxidation.^38^ Namely, PFOA sorption appeared substantial only in the solution where the mediator was present. This observation indicates that the sorption of PFOA may rely on specific side chain interactions or charges that are exposed upon oxidation of TvL, a mechanism previously reported to affect the sorption of PFOA to proteins.^37^ Comparatively, sorption of PFOS seems to be unaltered upon enzyme oxidation, perhaps because the interaction is driven by a side chain that remains structurally intact, and due to the hydrophobicity of PFOS.

## Environmental Implications

Although attractive as a biobased treatment method for PFAS remediation, the LMS appears to be unable to detectably degrade the perfluorinated compounds PFOA and PFOS. We were unable to observe transformation with the high-redox species TvL, despite the multiple literature reports of contaminant degradation by TvL and HBT systems^15,29^ and despite our own success with transforming the proxy compound CBZ. Investigation of the system by EPR analysis confirmed the generation of the expected radical species; however, no transformation of the target PFAAs was apparent across multiple treatment conditions.

Close examination of the mass of PFOA and PFOS contained in the reactors indicated that sorption of the substrate to the protein created an artifact that mimicked substrate loss under the treatment conditions. Variations in PFOA sorptive removal with and without the mediator suggest changes in enzyme conformation or residue exposure may alter protein–PFOA interactions; for PFOS, however, the affinity for the enzyme appears unaltered regardless of the presence or absence of the mediator. This result suggests that a host of specific and nonspecific interactions play a role in PFAA removal by the TvL protein.

The adsorption of PFAS to proteins is well-known and may be a viable remediation strategy in and of itself, although the quantity of enzyme needed may be prohibitive. However, the study of PFAS–protein binding mechanisms could provide insight into bioinspired adsorptive materials to selectively target the removal of PFAS from complex waste streams. Given the unique physicochemical properties and often surprising behavior of PFAS in aqueous matrices, it is particularly important for researchers to use rigorous controls when analyzing treatment and removal strategies with novel bioinspired technologies.

## Supporting Information Available

Details on additional experiments, analytical methods including LC-MS/MS, and data tables associated with the main text figures. The Supporting Information is available free of charge at https://pubs.acs.org/doi/10.1021/acs.estlett.3c00173.
