# Supplementary material for: An Artifact of Perfluoroalkyl Acid (PFAA) Removal Attributed to Sorption Processes in a Laccase Mediator System
Source: Environ Sci Technol Lett. 2023 Mar 24;10(4):337–42. doi: 10.1021/acs.estlett.3c00173 (PMC10100556; doi:10.1021/acs.estlett.3c00173)
Supplement: Supplementary file 1 — ez3c00173_si_001.pdf [file ez3c00173_si_001.pdf]

Supporting Information

An artifact of perfluoroalkyl acid (PFAA) removal attributed to sorption processes in a laccase-mediator system

Sophia D. Steffens[a], Edmund H. Antell[b], Emily K. Cook[b], Guodong Rao[c],

R. David Britt[c], David L. Sedlak[b], Lisa Alvarez-Cohen[b]*

[a] Department of Chemistry, University of California, Berkeley, CA, 94720, United States

[b] Department of Civil and Environmental Engineering, University of California, Berkeley, CA, 94720, United States

[c] Department of Chemistry, University of California, Davis, CA, 95616, United States

*Email: lisaac@berkeley.edu

Supporting Information

Text S1. Extraction protocol for enzyme-buffer solution and reactor vials……………………...S2

Text S2. LC-MS/MS Analytical Method……………………………………………………...S3

Text S3. EPR Experiment Solution Preparation………………………………….……….…..S4

Text S4. Corrected concentration calculations for enzyme-mediator screening……………..…..S4

Text S5. Enzyme Activity Assay………………………………………………………….……..S5

Table S1. PFAS Analytes Quantified by LC-MS/MS Analysis…………………….…..………..…S7

Table S2. PFOA mass recovered in subsamples versus extracted of protein solution + vials......S8

Table S3. PFOS mass recovered in subsamples versus extracted of protein solution + vials…...S8

Figure S1. Nitroxyl mediator compounds…………………..……………………………….….…S9

Figure S2. PFOS concentrations in enzyme-mediator screening experiments…………………..S9

Figure S3. CBZ treated with TvL and HBT – Enzyme activity and CBZ removal………..……S10

Figure S4. PFOA treated with TvL and HBT……………………………………...…..…S10

Figure S5. Enzyme activity in $CuSO_4$, acetate, and $FeSO_4$ solutions…………..………...……..S11

Figure S6. Oxidation of 1-hydroxybenzotriazole (HBT) to benzotriazole-*N*-oxyl (BTNO)…...S11

Figure S7. EPR spectra in $CuSO_4$ and acetonitrile with addition of PFOA, PFOS……………S12

Figure S8. Enzyme activity monitored in PFOA, PFOS reactors with TvL + HBT……...……S12

Text S1. Extraction Protocol for enzyme-buffer solution and reactors

To differentiate the mass loss of perfluorooctanoic acid (PFOA) and perfluorooctane sulfonic acid (PFOS) in solution between sorption to the *Trametes versicolor* laccase (TvL) enzyme and the reactor container (glass scintillation vial), an experiment was performed in which the reactor solution and glass vial were 'extracted' separately with basic MeOH. The reactor solutions were prepared in triplicate in the following manner: to glass scintillation vials (20 mL capacity), 5 mL of 10 mM $CuSO_4$ solution was added. Then, an aliquot of concentrated perfluoroalkyl acid (PFAA) stock (100-200 µL ranging from 2.5-250 µM PFOA or PFOS) was added via volumetric pipette. Then, a 60 mg portion of TvL enzyme (powder form, Millipore Sigma) was added; reactors with 2 mM HBT in CH_3CN and TvL only were tested. The vials were capped and placed in the shaker incubator for 24 hours (30ºC, 130 rpm).

Upon reactor setup and at the 24-hour time point, a 200 µL **Subsample** was taken from the bulk reactor solution and added directly to 200 µL of basic methanol (0.5% NH_4OH in MeOH). The subsample was pipetted from the middle of the TvL containing solution as some of the solid enzyme had settled to the bottom of the reactor. Then, the remaining TvL solution (~4.6 mL) was pipetted into a fresh vial; the same pipette tip was used to add 4.6 mL of basic methanol (0.5% NH_4OH) to the vial to dilute the solution 2x (**TvL solution extract**). To the original reactor (now emptied of TvL solution), 2 mL of 10 mM Cu_2SO_4 and 2 mL of basic MeOH were added to match the extraction matrix for the reactor solution; the added volume was used to rinse the original reactor container and re-dissolve any PFAA mass that may have sorbed to the glass over the course of 24 hours (**Reactor Extract**).

After letting the vials containing the basic MeOH/$CuSO_4$ solution rest on the benchtop for an additional 24 hours, samples from the **TvL Solution Extract** and **Reactor Extract** were

taken (1 mL into a 1.5 mL microcentrifuge tube) for the next stage of sample preparation. To quantify the PFAAs in the **Subsamples**, the **TvL Solution Extract**, and the **Reactor Extract**, the samples were subjected to the following procedure. First, the samples were vortexed for 5 minutes. Then, samples were centrifuged (10 minutes, 22ºC, 15,000 *g*) to remove $Cu(OH)_2$ that precipitated upon addition of the basic MeOH. From the centrifuged sample, 250 µL of volume was removed and diluted further in MeOH (*2x*) for quantification by LC-MS/MS.

Text S2. Liquid Chromatography Tandem Mass Spectrometry (LC-MS/MS) Analytical Methods

Per- and polyfluoroalkyl substance (PFAS) analytes were quantified by LC-MS/MS equipped with electrospray ionization in negative mode (Triple Quad 6460A, Agilent Technologies) multiple reaction monitoring (MRM). Gas and Sheath Gas Heater temperatures were 325 and 350°C, respectively. Gas and sheath gas flows were 9 L/min. The nebulizer was kept at 25 psi and the capillary voltage was 3.5 kV. Samples were prepared for analysis by dilution in methanol to a target concentration of 10 µg/L to ensure quantification within the calibration range (0.2-10 µg/L). The isotope dilution method was used to account for any potential matrix effects. Mass labeled [13C]- PFAAs (2-5 µg/L) were added to LC-MS/MS sample vials for each sample in the final stage of sample preparation. Analytes were separated using a Zinc-Diol guard column coupled to Zorbax C18 XDB guard and analytical columns (Agilent Technologies). The mobile phase (0.4 mL/min) was 5 mM ammonium acetate in water (A) and 5 mM ammonium acetate in methanol (B) with a solvent gradient: hold 0-2 min 95% A, ramp to 10% A by 10 min, hold 10-11.5 min 10% A, ramp to 95% A by 12 min, hold 12-18 min 95% A. The LC-MS/MS setup included a delay C18 column after the purge valve to decrease the

effect of possible contamination from upstream polytetrafluoroethylene (PTFE) components. PFAS were quantified by the transitions and collision energies listed in Table S1. Mobile phase blanks were run every 10 samples and select calibration samples were re-run every 20 samples to prevent contamination or carryover in the analysis.

Text S3. EPR Experiment Solution Preparation

The EPR sample of the benzotriazole-*N*-oxyl (BTNO) radical generated by the oxidation of HBT by Ce (IV) (see Supporting Information Figure S11) in CH_3CN was prepared by mixing 100 µL of 20 mM Cerium (IV) ammonium nitrate in CH_3CN and 100 µL of 20 mM HBT in CH_3CN. The spectrum was recorded at room temperature, using the following spectrometer settings: conversion time = 120 ms, modulation frequency = 100 kHz, modulation amplitude = 0.025 mT, and simulated using previously reported parameters (Galli et al. 2008). EPR samples of BTNO generated by oxidation of HBT by laccase enzymes were prepared by incubating aqueous solutions containing 1 mM HBT, 12 mg/mL TvL, and 10 µM $CuSO_4$. The spectra were recorded at 50 K using the following spectrometer settings: conversion time = 120 ms, modulation frequency = 100 kHz, modulation amplitude = 0.5 mT.

Text S4. Corrected concentration calculations for enzyme-mediator screening

In the preliminary enzyme-mediator screening conditions, summarized in Figure S2, we observed increases in PFOS concentration across all the treatment conditions that we attributed to evaporation through filter caps over the 28-day treatment period. Initial solution volumes were 10 mL contained in 30 mL polypropylene flasks. To correct for evaporation, we compared the detected concentrations ($C_{detected\text{-}Day\ X}$) in the treatment reactors between sampling timepoints to

the concentration in the control reactor ($C_{control\text{-}Day\ X}$), which contained no enzyme and no mediator. Specifically, $C_{detected\text{-}Day\ X}$ in each treatment reactor for Day 0, 7, 14, 21, 28 was divided by the quotient of the concentrations in the control on Day 0 and Day 0, 7, 14, 21, 28 to give $C_{corrected\text{-}Day\ X}$:

$$C_{corrected - Day\,X} = \frac{C_{detected - Day\,X}}{C_{control - Day\,0} \div C_{control - Day\,X}}$$

Additionally, to the control reactor set, 'doses' of 10 mM $CuSO_4$ buffer and CH_3CN were added weekly at the same time as enzyme (in 10 mM $CuSO_4$) and mediator (in CH_3CN) to maintain as consistent of volume addition to all the reactors as possible.

Text S5. Enzyme Activity Assay

Laccase activity was quantified by monitoring enzymatic conversion of 2,6-dimethoxyphenol (DMP) to the corresponding 3,5,3’5’-tetramethoxydiphenoquinone (Mizuno et al. 2009). The absorbance spectrum of the quinone was measured on a Shimadzu UV-vis spectrophotometer plate reader at a wavelength maximum of 468 nm (ε = 49,600 $M^{-1}cm^{-1}$) in a citrate-phosphate buffer (pH 3.8) prepared from a 0.1 M solution of citric acid and a 0.2 M solution of Na_2HPO_4. Assays were conducted in 96-well plates at a volume of 200 µL total. Buffer solution was added to the plate followed by the laccase containing solution; DMP was then quickly added and then the assay plate was placed in the plate reader. The laccase containing solution was diluted appropriately (1,000 - 10,000*x*) to achieve an absorbance in the range of 0.1-1 absorbance units (AU) for best accuracy. Absorbance was measured continuously over 10 minutes. Solutions were assayed in triplicate from which the average absorbance measurements were calculated. Absorbance data was related to enzyme activity using the Beer-Lambert law,

$$A = \varepsilon bC$$

where $\varepsilon = molar\ extinction\ coefficient, b = path\ length, and\ C = concentration.$ Path length was calibrated to 1 cm from a matrix blank using Gen5 Version 3.02 processing software. The change in concentration of substrate per minute (μmol/minute) was used to determine the enzyme activity, adjusted for dilution. The following equation was used,

$$Activity\left(\frac{U}{mL}\right) = \frac{\Delta A}{\varepsilon} \times 10^6 \times \frac{1}{D}$$

with a factor of 10^6 to convert from mol of product to μmol of product, and a factor of 1/D to correct for total dilution from the original enzyme containing solution.

Table S1. PFAS compounds analyzed via LC-MS/MS.

Compound	Internal Standard	Precursor Ion	Product Ion	Fragmentor Voltage (V)	Collision Energy (V)	Polarity
MPFBA	-	217	172	50	5	Neg
MPFPeA	-	266	222<br>221	60	2	Neg
MPFHxA	-	315	270	60	5	Neg
MPFOA	-	417	372	70	2	Neg
MPFNA	-	468	423	70	5	Neg
MPFHxS	-	403	103	150	40	Neg
MPFOS	-	503	80	190	60	Neg
PFBA	MPFBA	213	169	50	2	Neg
PFPeA	MPFPeA	263	219<br>68.9	60<br>92	2<br>8	Neg
PFHxA	MPFHxA	313	269<br>119	80	2<br>15	Neg
PFHpA	MPFOA	363	319<br>169	80	2	Neg
PFOA	MPFOA	413	369<br>169	80	3<br>14	Neg
PFNA	MPFNA	463	419<br>219	80	2<br>15	Neg
PFBS	MPFHxS	299	99<br>80	120	30<br>70	Neg
PFHxS	MPFHxS	399	99<br>80	125	50<br>80	Neg
PFOS	MPFOS	499	99<br>80	122	50<br>80	Neg
FHxSA	MPFOS	398	78	125	36	Neg
FOSA	MPFOS	498	78	125	36	Neg
6-2 FtS	MPFOS	427	407<br>80	140	25<br>35	Neg
AmPr-FHxSA	MPFOS	485	85	135	30	Pos

Table S2. PFOA mass recovery (mass detected in Subsample, Solution extract, Reactor extract, and Δ Solution, calculated as the difference in mass detected in the subsample at 0h and 24h) compared to total extracted mass. Summarized in Figure 2a of main text.

PFOA		Mass Recovered	Error
**TvL + HBT**			
	Subsample (24 hours)	82%	1.7%
	Solution extract	90%	1.2%
	Reactor extract	10%	0.6%
	Δ Solution	13%	6.1%
**TvL**			
	Subsample (24 hours)	92%	1.8%
	Solution extract	98%	1.3%
	Reactor extract	2%	0.1%
	Δ Solution	4%	2.6%%
**Control**			
	Subsample (24 hours)	87%	3.6%
	Solution extract	89%	0.6%
	Reactor extract	11%	1.2%
	Δ Solution	2%	4.4%

Table S3. PFOS mass recovery (mass detected in Subsample, Solution extract, Reactor extract, and Δ Solution, calculated as the difference in mass detected in the subsample at 0h and 24h) compared to total extracted mass. Summarized in Figure 2b of main text.

PFOS		Mass Recovered	Error
**TvL + HBT**			
	Subsample (24 hours)	66%	3.5%
	Solution extract	86%	3.3%
	Reactor extract	14%	1.6%
	Δ Solution	20%	4.5%
**TvL**			
	Subsample (24 hours)	65%	3.1%
	Solution extract	88%	2.5%
	Reactor extract	12%	0.4%
	Δ Solution	19%	4%
**Control**			
	Subsample (24 hours)	73%	16.5%
	Solution extract	60%	13.5%
	Reactor extract	40%	12.1%
	Δ Solution	8%	19.0%

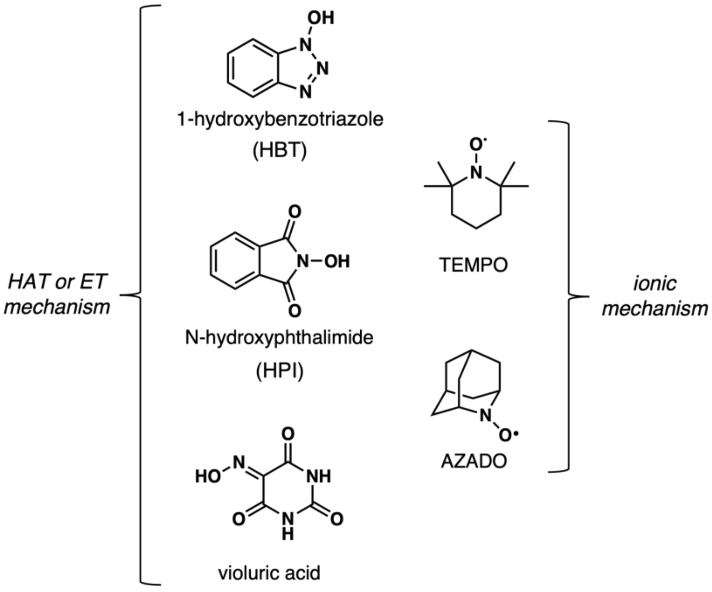

Figure S1. Nitroxyl (N-OH) mediators selected for screening. HBT, HPI, and violuric acid typically perform electron transfer by hydrogen atom transfer (HAT) or electron transfer (ET) mechanism; TEMPO and AZADO typically perform electron transfer by an ionic mechanism.

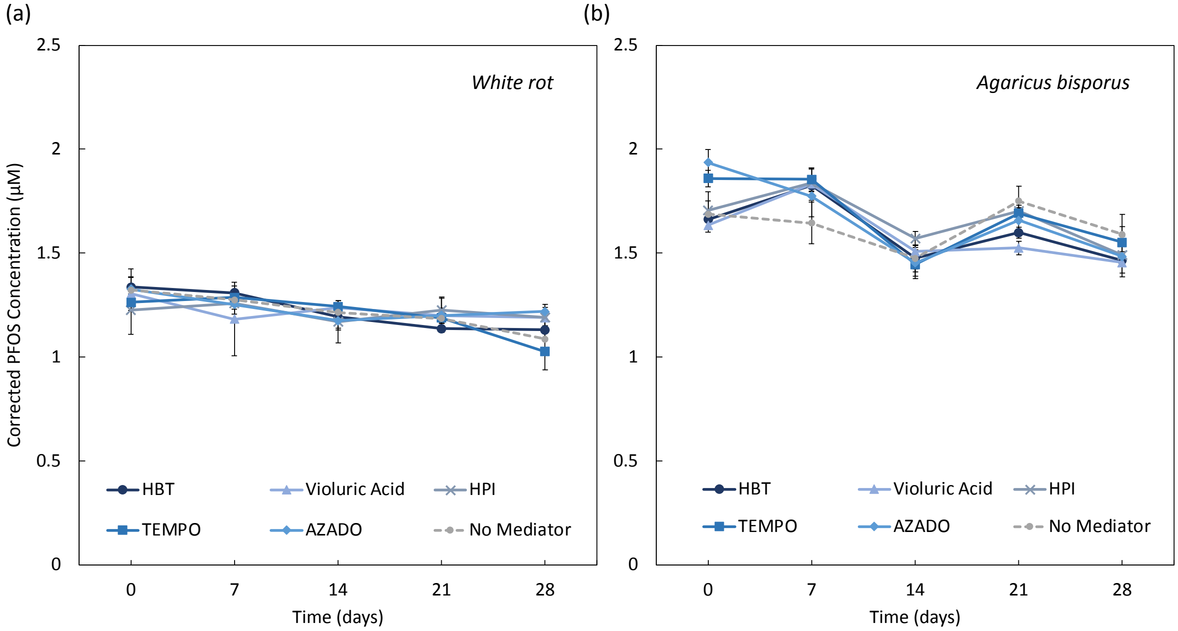

Figure S2. Screening of laccase-mediator combinations for reactivity towards PFOS. Laccase (1 U/mL) and mediator (20 µM) were added weekly. PFOS concentrations in the a) Native White Rot treated reactors and b) *Agaricus bisporus* treated reactors do not decrease significantly compared to the No Mediator control over the 28-day period ($p = 0.58$ White rot, $p = 0.24$ *Agaricus bisporus*). Concentrations were corrected for evaporation by comparison to an untreated reactor (details in Text S4). Error bars are the standard deviations of triplicate reactors.

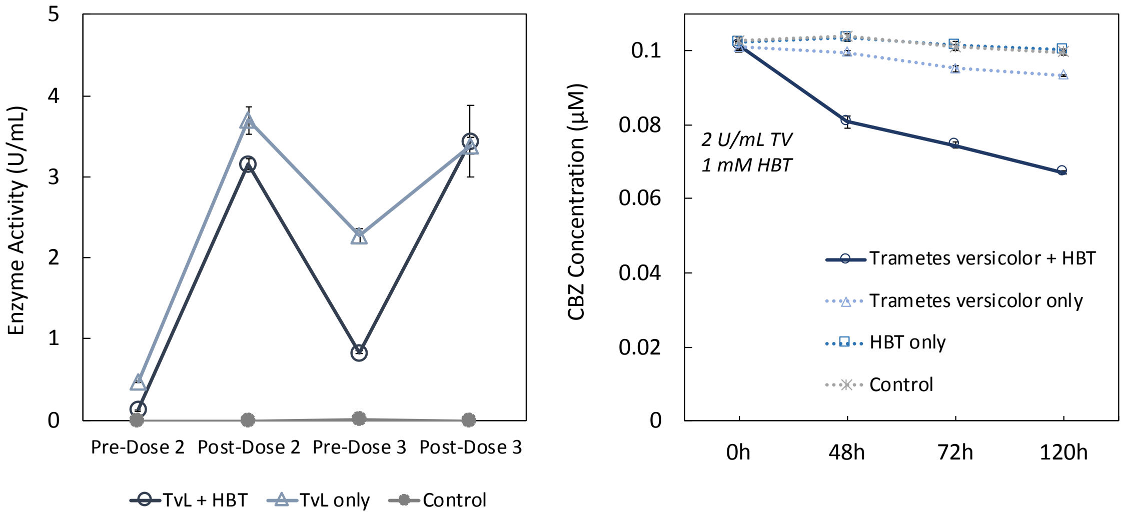

Figure S3. CBZ reactors treated with 2 U/mL TvL and 1 mM HBT in a 50 mM sodium malonate buffer (adjusted to pH 4.5). Doses of enzyme and mediator were added at 0 hours, 48 hours, and 72 hours (3 doses total). a) TvL activity monitored between doses by the DMP assay; b) CBZ concentration over the course of the treatment. Error bars represent the standard deviations of concentrations measured in triplicate reactors.

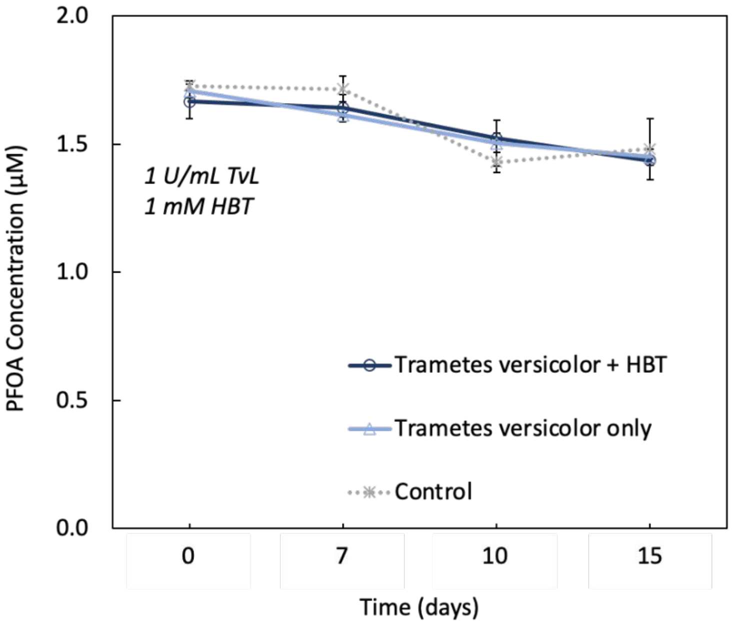

Figure S4. PFOA concentration in a 50 mM sodium malonate buffer (adjusted to pH 4.5). Doses of 1 U/mL TvL and 1 mM HBT were added every 2-3 days (6 doses total). Error bars are the standard deviations of concentrations measured in triplicate reactors.

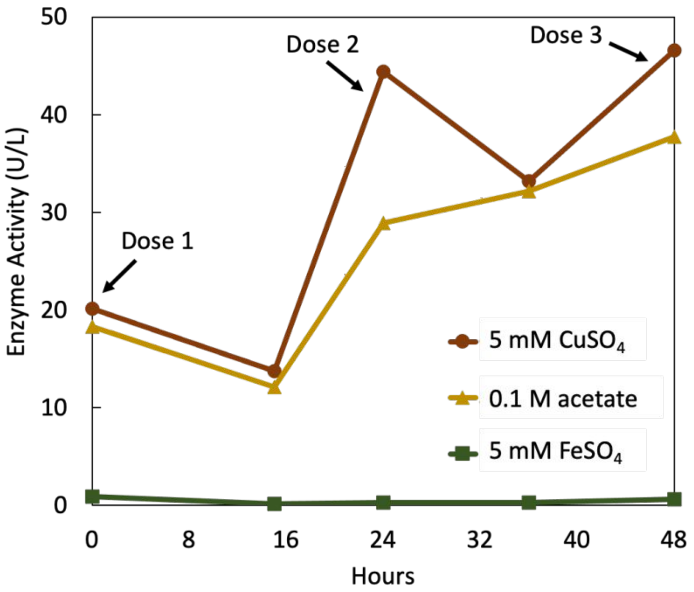

Figure S5. Monitoring *Trametes versicolor* activity in three different solutions indicated that enzyme activity was retained in 5 mM $CuSO_4$ solution and 0.1 M sodium acetate buffer; no enzyme activity was observed in 5 mM $FeSO_4$ solution. Details of activity assay in Text S5.

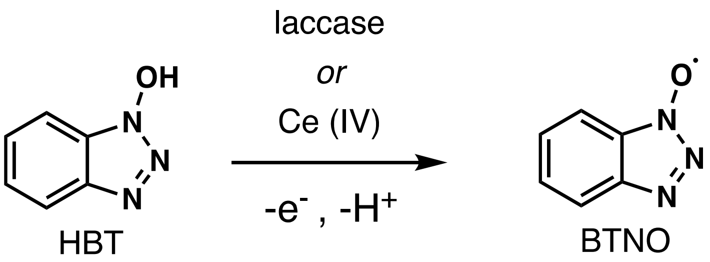

Figure S6. Oxidation of 1-hydroxybenzotriazole (HBT) to the radical species benzotriazole-*N*-oxyl (BTNO) by electron and proton transfer. The oxidation can be carried out by Ce (IV) ammonium nitrate in CH_3CN in an equimolar ratio, or by laccase in an aqueous buffer solution.

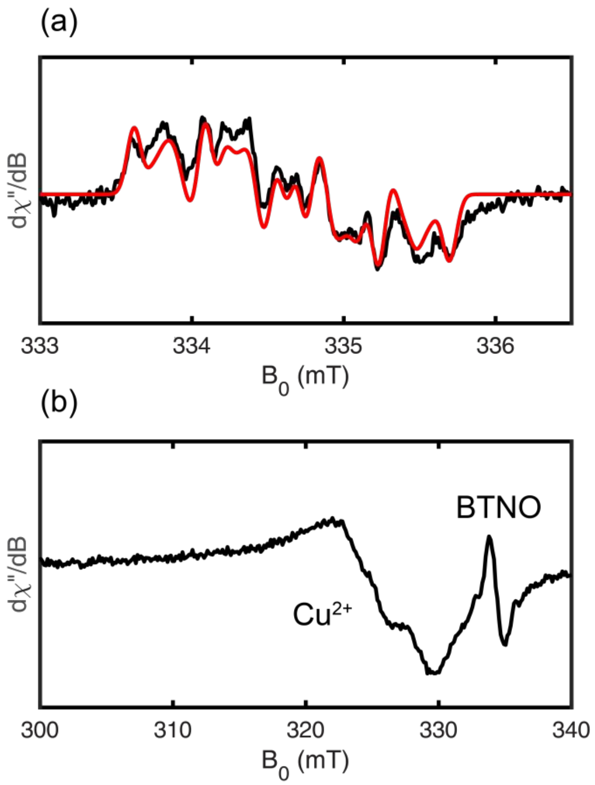

Figure S7. X-band CW EPR spectra. (a) Room temperature EPR spectrum of BTNO radical generated in CH_3CN (black trace) and simulation (red trace). Simulation parameters: $g = 2.0069$, three ^{1}H with a (^{1}H) = 1.0, 5.7 and 12.8 MHz, three ^{14}N a (^{14}N) = 1.6, 4.3 and 13.3 MHz. (b) EPR spectrum of BTNO radical generated in the laccase system. The feature on the left is due to Cu^{2+} EPR signal.

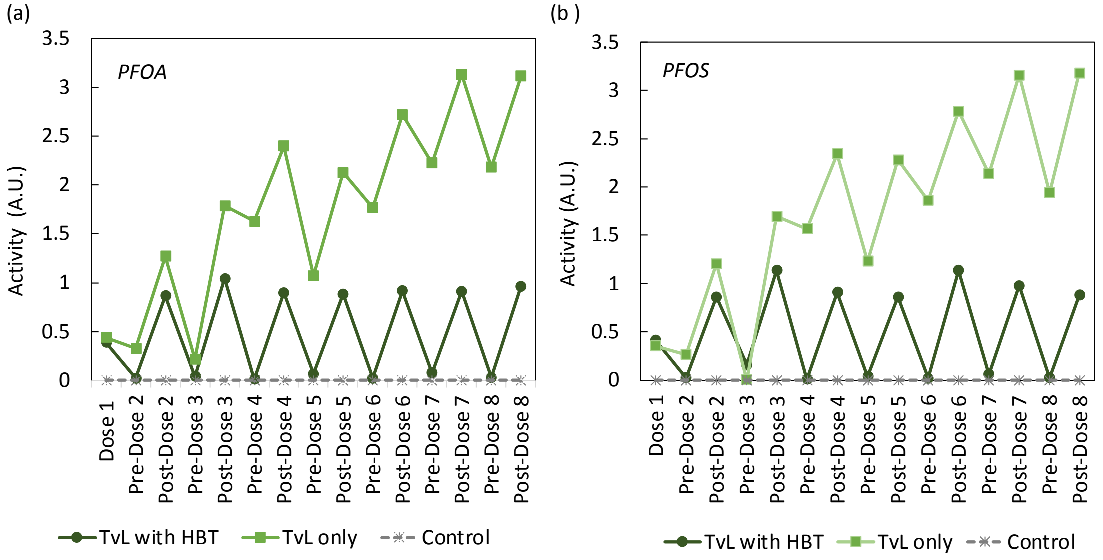

Figure S8. Enzyme activity monitored by the DMP assay over the course of the reaction in reactors containing 0.1 µM PFOA or PFOS in 10 mM $CuSO_4$ treated with ~1 U/mL TvL and 1 mM HBT. Doses were added twice daily, with approximately 6-8 hours in between doses.

References

1. Mizuno, H.; Hirai, H.; Kawai, S.; Nishida, T. Removal of Estrogenic Activity of Iso-Butylparaben and n-Butylparaben by Laccase in the Presence of 1-Hydroxybenzotriazole. *Biodegradation* **2009**, *20* (4), 533–539.

2. Galli, C.; Gentili, P.; Lanzalunga, O.; Lucarini, M.; Pedulli, G. F. Spectrophotometric, EPR and Kinetic Characterisation of the >N-O* Radical from 1-Hydroxybenzotriazole, a Key Reactive Species in Mediated Enzymatic Oxidations. *Chem. Commun.* **2004**, *20*, 2356–2357.
